# Profiling of Carnitine Shuttle System Intermediates in Gliomas Using Solid-Phase Microextraction (SPME)

**DOI:** 10.3390/molecules26206112

**Published:** 2021-10-10

**Authors:** Joanna Bogusiewicz, Katarzyna Burlikowska, Karol Jaroch, Paulina Zofia Gorynska, Krzysztof Gorynski, Marcin Birski, Jacek Furtak, Dariusz Paczkowski, Marek Harat, Barbara Bojko

**Affiliations:** 1 Department of Pharmacodynamics and Molecular Pharmacology, Faculty of Pharmacy, Collegium Medicum in Bydgoszcz, Nicolaus Copernicus University in Torun, 85-089 Bydgoszcz, Poland; j.bogusiewicz@cm.umk.pl (J.B.); k.burlikowska@cm.umk.pl (K.B.); karol.jaroch@cm.umk.pl (K.J.); gorynska@cm.umk.pl (P.Z.G.); gorynski@cm.umk.pl (K.G.); 2 Department of Neurosurgery, 10th Military Research Hospital and Polyclinic, 85-681 Bydgoszcz, Poland; mbir-ski@poczta.fm (M.B.); jacek.furtak2019@gmail.com (J.F.); darek_paczkowski@vp.pl (D.P.); harat@10wsk.mil.pl (M.H.); 3 Department of Neurosurgery and Neurology, Faculty of Health Sciences, Collegium Medicum in Bydgoszcz, Nicolaus Copernicus University in Torun, 85-168 Bydgoszcz, Poland

**Keywords:** glioma, cancer, carnitine, acylcarnitine, solid-phase microextraction SPME, liquid chromatography–mass spectrometry LC–MS

## Abstract

Alterations in the carnitine shuttle system may be an indication of the presence of cancer. As such, in-depth analyses of this pathway in different malignant tumors could be important for the detection and treatment of this disease. The current study aims to assess the profiles of carnitine and acylcarnitines in gliomas with respect to their grade, the presence of isocitrate dehydrogenase (IDH) mutations, and 1p/19q co-deletion. Brain tumors obtained from 19 patients were sampled on-site using solid-phase microextraction (SPME) immediately following excision. Analytes were desorbed and then analyzed via liquid chromatography–high-resolution mass spectrometry. The results showed that SPME enabled the extraction of carnitine and 22 acylcarnitines. An analysis of the correlation factor revealed the presence of two separate clusters: short-chain and long-chain carnitine esters. Slightly higher carnitine and acylcarnitine concentrations were observed in the higher-malignancy tumor samples (high vs. low grade) and in those samples with worse projected clinical outcomes (without vs. with IDH mutation; without vs. with 1p/19q co-deletion). Thus, the proposed chemical biopsy approach offers a simple solution for on-site sampling that enables sample preservation, thus supporting comprehensive multi-method analyses.

## 1. Introduction

Gliomas are among the most dangerous and insidious brain tumors due to their high heterogeneity and the late manifestation of a wide range of non-specific symptoms, such as seizures, headaches, nausea, dizziness, fatigue, vision problems, and numbness [1,2,3,4,5]. Delayed diagnosis favors tumor progression and leads to worse prognoses and, consequently, a rapid decrease in the patient’s quality of life. As a consequence, the introduction of accurate medical interventions, which often combine neurosurgery and chemo- or radiotherapy, is necessary. The selection of the best treatment is mainly based on the combined results of a histopathological examination and genetic and immunochemical testing. Genetic testing—for example, those that analyze the status of O6-methylguanine-DNA methyltransferase (MGMT) methylation, the presence of IDH mutation, or 1p/19q co-deletion—serves as a complement to the diagnosis process and enables accurate clinical prognoses [2,5]. Nonetheless, the survival rate of glioma patients is still low due to a lack of effective treatment methods [1,2,3,4,5]. Thus, it is critical to further expand our basic knowledge about the metabolism of these tumors, as such information is indispensable in improving clinical prognoses and the effectiveness of treatments.

Cancer cells are characterized by increased metabolism, which generates ATP, NADPH, and other intermediates for tumor growth, as well as high adaptability to the dynamically changing microenvironment [3,6]. For a long time, the so-called Warburg effect—wherein increased aerobic glycolysis results in enhanced lactate production, rather than pyruvate production—has been cited as the main source of energy production in gliomas and other neoplasms [6]. However, recent findings suggest that altered fatty acid oxidation (FAO) is also an important marker of glioma initiation and development [3,6,7,8]. This metabolic pathway is mainly regulated by the carnitine shuttle system, which consists of enzymes and protein transporters that are responsible for transporting fatty acids through the mitochondrial membrane [6]. Although the expression and enzymatic activity of this pathway’s protein components (e.g., carnitine: acylcarnitine translocase (CACT), carnitine palmitoyltransferase I (CPT-1) or carnitine palmitoyltransferase II (CPT-2), and long-chain acylocarnitines dehydrogenase (LCAD)) have been extensively studied [3,6], the role and fate of carnitine and the esters (also known as acylcarnitines) produced from transporting fatty acids though the mitochondrial membrane remain unclear.

There are many methods that can be used to analyze changes in the chemical composition of tissues, such as homogenization followed by liquid–liquid extraction, microdialysis, and solid-phase microextraction (SPME) [9,10,11,12]. SPME, which is based on the interaction between an active sorbent and targeted substances dispersed in a given matrix, offers a number of significant advantages. One of the most notable of these advantages is SPME’s ultra-simple sampling procedure, which enables samples to be acquired directly from tissues without any major structural disruption. In addition, the SPME protocol can be implemented on-site (i.e., surgery room) by medical personnel who have no analytical background. The most common SPME device is a thin nickel–titanium fiber coated with an active sorbent to a final diameter of ca. 250 μm. Under the SPME protocol, the probe is first introduced into the tissue for a time period that has been predetermined to enable optimal metabolite binding, followed by desorption of the analytes from the device into an organic solvent. It should be emphasized that the extracted analytes do not require any additional treatment prior to instrumental analysis on an LC–MS platform. This methodology has been characterized in greater detail in the literature [9,13].

Our previous work on the untargeted metabolomic characterization of brain tumors found characteristic differences in metabolome composition with regards to histological type and genetic aberrations [13,14,15]. However, (semi)quantitative targeted analysis would provide more accurate information about the biochemical changes in a cancerous tissue. Therefore, this study aims to develop a more in-depth understanding of the intermediates in a carnitine shuttle system using data acquired from untargeted lipidomic analyses of brain tumors via SPME–LC–MS, with particular consideration given to tumor grade, the presence of IDH mutation, and 1p/19q codeletion.

## 2. Results

The use of SPME–LC–HRMS for acylcarnitine analysis enabled identification of carnitine (C) and 22 simple-chain saturated and unsaturated acylcarnitines (AC) (Table 1): five short-chain acylcarnitines (AC C2–AC C5, SCAC), seven medium-chain acylcarnitines (AC C6–AC C12, MCAC), and ten long-chain acylcarnitines (AC C14–AC C20, LCAC). SPME extraction from intact tissue did not cause any damage to the collected tumor, which precluded further performance of routine tests, i.e., histological or genotyping. Our findings showed that the level of carnitine (C) was significantly higher in high-grade gliomas (HGG) when compared to low-grade gliomas (LGG) with a ratio of 4.21, and in IDH wild-type (IDHw) compared to IDH mutant (IDHm) tumors, with fold change 3.91. Although no statistically significant difference was observed in the tumor carnitine levels of patients with and without the presence of 1p/19q co-deletion, the area under the peak was higher for patients with tumors not featuring co-deletion (n-del:del ratio 3.37).

**Figure 1:**
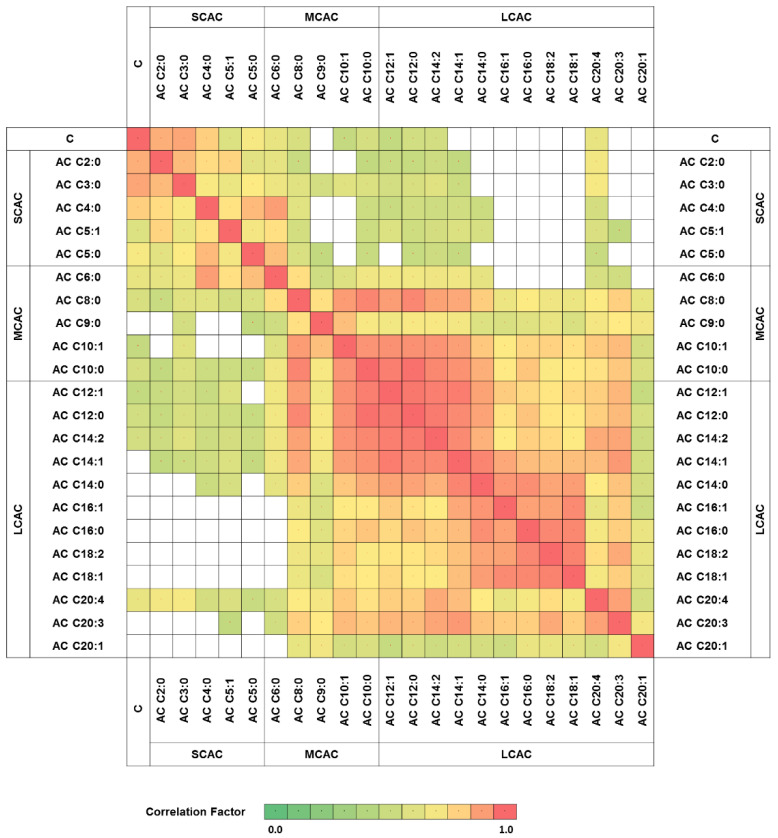
Carnitine and acylcarnitines correlation network. Only statistically significant correlation factors were presented (*p* < 0.05). AC—acylcarnitine; C—carnitine; SCAC—short-chain-length acylcarnitines; MCAC—medium-chain-length acylcarnitines; LCAC—long-chain-length acylcarnitines.

To explore the relationship between carnitine and its particular esters in all of the obtained glioma samples, correlation clustering analysis was performed The results revealed a high correlation factor for specific patterns of acylcarnitines (Figure 1). The analytes classified as SCAC represented one correlation cluster, with a correlation coefficient above or equal to 0.63, while MCAC and LCAC were correlated with each other, with a minimum factor of 0.51. Moreover, no clear correlation was observed between the metabolites with short- and long-acyl-chain analytes (Figure 1).

**Figure 2:**
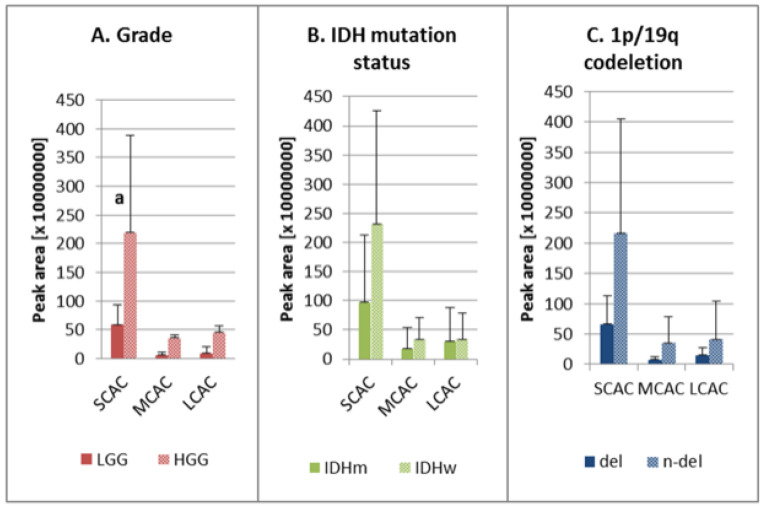
Ratios of SCAC, MCAC, and LCAC in the studied groups: (**A**) low-grade (LGG) and high-grade glioma (HGG); (**B**) IDH mutation status—IDH mutant (IDHm) and IDH wild-type (IDHw); (**C**) 1p/19q co-deletion—presence of deletion (del) and absence of deletion (n-del). AC—acylcarnitine; del—presence of 1p/19q co-deletion; HGG—high-grade glioma; IDHm—IDH mutation, IDHw—IDH wild-type; LCAC—long-chain-length acylcarnitine; LGG—low-grade glioma; MCAC—medium-chain-length acylcarnitines; n-del—absence of 1p/19q co-deletion; SCAC—short-chain-length acylcarnitines; ^a^ the average peak area for HGG is statistically significantly different from LGG, *p* < 0.05.

The analysis of acylcarnitines with different acyl chain groups showed that the average peak areas for SCAC, MCAC, and LCAC were larger for HGG than for LGG, although statistical significance was observed only for SCAC (Figure 2A). Our findings revealed also higher levels of these analytes in the IDH wild-type samples (versus the mutants), although the difference was not statistically significant (Figure 2B). Similarly, the peak areas for all acylcarnitines were not significantly higher in the samples without the chromosomal aberration (Figure 2C).

In the next step, comparative analysis of the acylcarnitine profiles in the different subgroups of the glioma samples was conducted. The groups considered cancer grade, IDH mutation status, and the presence of 1p/19q co-deletion. A detailed list of calculated values in all studied samples is given in Table S1. The analysis revealed that a statistically significant difference between HGG and LGG was observed for AC C3:0, AC C9:0, AC C10:1, AC C14:2, and AC 20:3, with peak areas over two times higher in higher-grade lesions (Table 1). Moreover, the findings showed a higher level of AC C12:0 in the IDH wild-type samples (versus the mutated samples) and a lower level of AC C16:1 (Table 1). With regards to 1p/19q codeletion status, it was observed that the peak area for AC C16:1 in the samples without codeletion was significantly lower than that in wild-type ones, and the ratio between the studied groups (n-del:del) was 0.59 (Table 1). A detailed description of the performed comparison is given in the Supplementary Materials (Table S2).

## 3. Discussion

The use of multiplatform studies combining various analytical methods—for example, extending routine tests to -omic studies—has become more common in basic cancer research [16], as such approaches are able to provide detailed analysis of the metabolic pathways and identify tumor vulnerability [17]. However, combining various techniques to analyze a single sample also faces limitations due to small amounts of the sample and analyte instability. Given these limitations, it is critical to continue to explore other approaches to sampling.

The methodology of profiling carnitine and its esters proposed herein is based on non-sample-consuming sampling, which is an approach that could enable additional testing of the obtained tissue (e.g., chemical biopsy followed by genetic testing or histopathology examination of the same specimen). Moreover, due to its simplicity, the sampling combined with the extraction of small molecules can be performed on-site (e.g., in the surgery room), which makes it radically different than other protocols used for tissue preparation prior to LC–MS analysis. Most of the currently used protocols include homogenization and multi-step (multi-solvent) solid–liquid extraction [18,19]. The goal of the current study was to verify if the extraction protocol dedicated to the untargeted screening of brain tumor lipidome enables the extraction of a representative range of carnitine derivatives and carnitine itself, thus observing dysregulation of small molecules in the carnitine shuttle system. Indeed, the results indicated that carnitine as well as its 22 short-, medium-, and long-chain acyl derivatives were extracted. The obtained data were then analyzed in the view of their biological significance and compared with up-to-date literature reports to verify findings. It needs to be emphasized here that the instrumental analysis was still performed in the university laboratory; however, the presented sampling approach can be combined directly with a mass spectrometer or other detector for fast quantitative analysis [20,21,22,23].

Carnitine is integral to the proper functioning of the enzymes (CPT-1, CPT-2, CACT) involved in transporting long-chain fatty acids across the inner mitochondrial membrane. Thus, this metabolite is considered a crucial regulator of the carnitine shuttle system [6,24]. Prior studies have examined levels of this metabolite in a variety of malignant neoplasms, including glioma, hepatocellular carcinoma, breast cancer, and prostate cancer, with findings showing higher concentrations in malignant tissues compared to histologically healthy samples [6,11,12,25,26]. In our study, we did not compare healthy and cancerous samples; rather, we compared cancerous samples with varying grades, with results indicating higher carnitine content in higher-grade tumors. Furthermore, our results agreed with a prior comprehensive metabolomic analysis of mutant and wild-type samples, which revealed higher carnitine concentrations in glioblastoma cells without the IDH mutation [24]. The higher levels of carnitine detected in HGG and IDHw could be related to the increased metabolism of these tumors compared to LGG and IDHm, which is consistent with Melone et al.’s [6] model of cancer metabolism. The carnitine shuttle system plays an important role in cancer plasticity, and it enables the metabolic demands of proliferating cancer cells to be fulfilled, even in adverse conditions.

The acylcarnitines identified in our study have also been observed by other researchers in their work on different types of malignant neoplasms (e.g., kidney, or liver); however, these prior works used sample preparation protocols that are more complex than SPME [11,27]. For instance, prior works have mainly used tissue homogenization, which is a sample-consuming approach, followed by liquid–liquid extraction prior to instrumental analysis. Thus, this method precludes the re-use of the sample with other diagnostic approaches. Moreover, the SPME approach used in the present work combines sampling and extraction into a single step, which greatly simplifies the sample preparation procedure. The utility of this non-sample-consuming technique for tissue analysis has been widely reported elsewhere [9,28,29,30].

The characteristic correlation clustering of SCAC and LCAC (in two separate clusters) was observed (Figure 1). The MCAC correlation clusters did not show clear patterns. Lu et al. [11] obtained similar results in their attempt to profile acylcarnitines in liver cancer, namely, that LCAC and SCAC formed separate correlation clusters. This phenomenon could be the result of the dependence of long-acyl-chain acylcarnitines on the enzymes in the carnitine shuttle system. Due to their small size, acylcarnitines with short acyl chains are able to pass through the mitochondrial membrane without the support of CPT-1, CPT-2, and LCAD, whereas the passage of LCAC is strictly controlled by the carnitine shuttle system [3]. The MCAC transport system is supposed to fall in between these two regulations.

The first of the studied factors was tumor malignancy grade. It was observed that the high malignancy status corresponded to a higher level of acylcarnitines (Table 1, Figure 2A). This could be explained by activated proliferation and the higher rates of metabolism in malignant lesions. The findings of Kant et al.’s [3] study of FAO in gliomas showed that glioblastomas contained higher levels of acylcarnitines compared to low-grade astrocytomas, which could be due to enhanced activity among carnitine shuttle enzymes [3].

One of the main prognostic factors with respect to gliomas is the presence of a mutation in the gene encoding isocitrate dehydrogenase (IDH) [2,5], which is the enzyme responsible for catalyzing the oxidative decarboxylation of isocitrate to 2-oxoglutarate in the tricarboxylic acid (TCA) cycle. During this process, nicotinamide adenine dinucleotide phosphate (NADP^+^) is reduced to nicotinamide adenine dinucleotide phosphate (NADPH) [31,32], which serves as a redox power for overcoming oxidative stress generated during cancerogenesis, as well as a co-enzyme in anabolic processes resulting in cellular proliferation. FAO by production of acetyl CoA, which is the TCA substrate, can be influenced by alterations related to IDH mutation [32]. This could explain our observations of higher levels of carnitine esters in wild-type samples (Figure 2B), but the changes in the levels of particular acylcarnitines are not consistent. Therefore, further investigation is needed to fully elucidate the data (Table 1). It is likely that IDH mutation leads to changes in the activity of the carnitine shuttle system, which in turn results in the observed metabolic changes. This assumption could help to improve the prognosis of IDHm patients, as the down-regulation of fatty acid transport reduces the proliferation rate of cancer cells and, ultimately, tumor malignancy [2,5,33]. A similar observation has been reported by Miyata et al. [24], who also identified lower concentrations of carnitine and acetylcarnitine in IDH-mutant gliomas compared to the wild-type variants. However, these reports are not consistent because Kant et al. [3] did not observe any major differences in the amount of detected carnitine and its esters.

Testing aimed at detecting the co-deletion of chromosomes 1p and 19q and IDH mutation is one of the main genetic approaches to glioma diagnosis, as it enables the differentiation of oligodendriogliomas from astrocytomas [2,5]. In our study, we did not observe significant changes in the acylcarnitine level between samples without and with 1p/19q codeletion, with the exception of AC 16:1 in which the normalized level was significantly lower in n-del samples (Table 1, Figure 2C). It was not possible to explain the direct biochemical correlation between lipid metabolism and the presence of 1p/19q co-deletion based on the available literature. However, it is worth mentioning that the patients without this aberration had poorer responses to radiotherapy and worse survival rates compared to patients with the 1p/19q co-deletion [34]. Therefore, slightly higher levels of carnitine intermediates in wildtype might help to explain different responses to radiotherapy among patients with diverse status of this aberration in the future.

The investigations presented herein demonstrated that the use of SPME sampling of intact brain tumors on-site followed by untargeted LC–HRMS analysis in the lab enabled carnitine and 22 of its esters to be profiled in glioma samples. The results of this study confirmed that alterations in the carnitine shuttle system might be an important factor in estimating glioma malignancy and assessing clinical prognosis. Our findings also revealed that SCAC and LCAC formed clearly separated correlation clusters, which could indicate their different levels of dependence on the carnitine shuttle system. Moreover, we were able to profile carnitine and acylocarnitines in glioma samples. We observed that the content of carnitine and acylcarnitines was usually higher in higher-malignancy tumors (HGG vs. LGG) or in patients with worse clinical outcomes (IDHw vs. IDHm and with 1p/19q co-deletion vs. without 1p/19q co-deletion). However, it was not possible to form any firm conclusions due to the high heterogeneity among the studied samples, small cohort, and the lack of results obtained using reference methods (e.g., using immunochemistry to assess enzyme activity). At the same time, despite these obvious limitations of the presented studies, the results indicated validity of further in-depth targeted quantitative analysis with the use of the proposed SPME sampling protocol and LC–MS/MS method. In the future, it may be possible to obtain more detailed information about the biology of brain tumors by combining in situ SPME sampling coupled to LC–MS/MS with histological, immunochemical, or genetic platforms. Moreover, in the view of the recent work which demonstrated that SPME enables spatially resolved analysis of the living human brain [35], it can be expected that one of the future directions in carnitine analysis will be their simultaneous profiling in cancerous and normal tissue.

## 4. Materials and Methods

### 4.1. Biological Material

Primary brain tumors were obtained via neurosurgical procedures conducted at the 10th Military Research Hospital and Polyclinic in Bydgoszcz, Poland. Overall, 19 samples were analyzed. The characteristics of these samples were as follows: 7 were low-grade gliomas (LGG) and 12 were high-grade gliomas (HGG); 10 were IDH mutant (IDHm) tumors, and 9 were IDH wild-type (IDHw) tumors; 7 featured 1p/19q co-deletion (del), while 12 did not (n-del). A detailed summary of the patients’ characteristics is presented in Table S3 in the Supplementary Materials.

The study was approved by the Bioethical Committee in Bydgoszcz, Poland (KB 628/2015).

The grades of studied tumors were assessed by a histopathologist. Grade 1 and 2 tumors were included in the low-grade glioma (LGG) group. Grade 3 and Grade 4 tumors were included in the high-grade glioma (HGG) group.

The IDH status and 1p/19q codeletion presence were assessed with the use of SALSA MLPA P088-D1 kit (MRC-Holland, Amsterdam, the Netherlands) according to the manufacturer’s protocol.

### 4.2. Chemical Biopsy (Solid-Phase Microextraction) Protocol

Sampling was conducted using 7 mm C18 fibers kindly provided by Supelco, Merck, immediately following the removal of the brain tumor. To this end, the protocol developed by Bogusiewicz et al. [12] was employed, with minor modifications. The fibers were preconditioned overnight in a methanol:water (1:1 *v*/*v*) solution to activate the sorbent and were rinsed with water directly before sampling in order to remove any organic solvent residue. The fibers were then inserted into the tumor tissue for 30 min and then rinsed again in water to remove any residues from cell debris or blood. The brain tumor sampling was performed at room temperature. To verify the potential impact of temperature on the stability of investigated compounds, the data obtained at room temperature were compared with the results of the extraction carried on ice (Table S4). The experiment was performed using fresh mouse brain as a model tissue. Five fibers were inserted in the area of the hypothalamus. Two of the metabolites showed significant differences.

After extraction, the fibers were stored in a freezer at −30 °C until desorption (1 h), which was conducted in silanized glass vials with 150 μL of an isopropanol:methanol (1:1 *v*/*v*) solution and agitation at 1200 rpm. Pooled quality control (QC) and extraction blanks (negative control) were also prepared.

### 4.3. Instrumental Analysis

The liquid chromatography-high resolution mass spectrometry (LC–HRMS), platform consisted of a Dionex UltiMate 3000 RS autosampler, a Dionex Ultimate 3000 RS pump (Thermo Fisher Scientific, Dionex, Bremen, Germany), and a QExactive Focus high-resolution mass spectrometer (Thermo Fisher Scientific, Bremen, Germany).

LC analysis was conducted using the following parameters: phase A—5 mM ammonium acetate in water; phase B—acetonitrile; gradient—0.0–2.0 min 96% B, 15.0 min 80% B, 15.1–21.0 min 96% B; SeQuantZIC-cHILIC—3 μm 100 × 2.1 mm column; flow—0.4 mL/min; oven temperature—40 °C; and injection volume—10 μL.

The present study used positive ion mode with the following parameters: a scan range of 100–1000 *m*/*z*; acquisition via AGC (1,000,000 ions); a spray voltage of 1.5 kV; an S-lens RF level of 55%; an S-lens voltage of 25 V; a skimmer voltage of 15 V; a capillary temperature of 325 °C; sheath gas at 60 a.u.; aux gas at 30 a.u.; spare gas at 2 a.u.; and a probe heater temperature of 320 °C. Only acylcarnitines in the extraction QC samples with a coefficient of variation (CV) of less than 10% were accepted for analysis. These acylcarnitines were identified by matching their fragmentation patterns with spectra libraries at a mass accuracy of <3 ppm (the presence of characteristic fragment: 85.0290 peak in MS/MS spectra). Full MS/dd-MS2 discovery mode was used for this purpose. Matching was conducted using the following fragmentation parameters: mass resolution—35000 FWHM; AGC target—2E4; minimum AGC—8E3; intensity threshold—auto; maximum IT—auto; isolation window—3.0 *m*/*z*; stepped collision energy—20 V, 30 V, 40 V; loop count—2; and dynamic exclusion—auto.

### 4.4. Data Processing and Statistical Analysis

Acylcarnitine identification was performed using LipidSearch 4.1.30 (Thermo Fisher Scientific, San Jose, CA, USA) software, which is capable of identifying simple-chain carnitine esters with eight or more carbons in their structure (AC C8:0) (Table S5 in the Supplementary Materials). As such, carnitine and acylcarnitines with shorter chains were searched manually using mzCloud and the Human Metabolome Database (HMDB).

Statistical analysis was conducted using Statistica 13.3 PL (StatSoft, Inc., Tulsa, OK, USA) software. The average peak area for all analytes was calculated, and statistical tests were applied. In particular, Levene’s test was used to assess variation, and the Shapiro–Wilk test was applied to assess normality. A T-test was subsequently applied when variation was homogenous and the variables were normal, while the Mann–Whitney U Test was used in all other cases.

For in-depth analysis of particular acylcarnitines, normalization on acylcarnitine groups was performed (SCAC, MCAC, LCAC).

## Figures and Tables

**Table 1:** Acylcarnitines identified in gliomas sampled via solid-phase microextraction (SPME). Table 1 represents identification details and ratios of normalized peak areas for detected analytes. AC—acylcarnitine; del—presence of 1p/19q co-deletion; HGG—high-grade glioma; IDHm—IDH mutation, IDHw—IDH wild-type; LCAC—long-chain-length acylcarnitine; LGG—low-grade glioma; MCAC—medium-chain-length acylcarnitines; n-del—absence of 1p/19q co-deletion; SCAC—short-chain-length acylcarnitines.

Group	AC	Chemical Formula [M + H^+^]	M/Z [M + H^+^]	RT [min]	HGG: LGG	IDHw: IDHm	n-del: del
**SCAC**	**AC C2:0**	C_9_H_18_O_4_N_1_	204.1231	12.46	0.87	0.97	0.93
	**AC C3:0**	C_10_H_20_O_4_N_1_	218.1387	11.00	2.89 ^a^	1.68	1.68
	**AC C4:0**	C_11_H_22_O_4_N_1_	232.1543	9.72	0.85	0.94	0.84
	AC C5:1	C_12_H_22_O_4_N_1_	244.1543	9.28	0.95	0.78	0.83
	**AC C5:0**	C_12_H_24_O_4_N_1_	246.1700	8.96	1.38	1.00	1.22
**MCAC**	**AC C6:0**	C_13_H_26_O_4_N_1_	260.1856	8.37	0.66	0.91	1.11
	**AC C8:0**	C_15_H_30_O_4_N_1_	288.2169	7.79	1.20	1.04	0.95
	**AC C9:0**	C_16_H_32_O_4_N_1_	302.2325	7.62	29.98 ^a^	2.12	1.37
	**AC C10:1**	C_17_H_32_O_4_N_1_	314.2326	7.52	3.86 ^a^	1.18	0.74
	**AC C10:0**	C_17_H_34_O_4_N_1_	316.2484	7.48	1.53	1.09	0.93
**LCAC**	**AC C12:1**	C_19_H_36_O_4_N_1_	342.2640	7.27	1.50	1.56	0.99
	**AC C12:0**	C_19_H_38_O_4_N_1_	344.2796	7.23	1.34	1.66 ^b^	1.22
	**AC C14:2**	C_21_H_38_O_4_N_1_	368.2797	7.12	2.72 ^a^	1.94	1.33
	**AC C14:1**	C_21_H_40_O_4_N_1_	370.2953	7.07	1.06	1.19	1.02
	**AC C14:0**	C_21_H_42_O_4_N_1_	372.3108	7.10	0.89	1.08	0.96
	**AC C16:1**	C_23_H_44_O_4_N_1_	398.3266	6.96	0.82	0.60 ^b^	0.59 ^c^
	**AC C16:0**	C_23_H_46_O_4_N_1_	400.3423	6.96	0.92	0.96	1.06
	**AC C18:2**	C_25_H_46_O_4_N_1_	424.3422	6.89	1.05	0.79	0.86
	**AC C18:1**	C_25_H_48_O_4_N_1_	426.3579	6.84	0.87	0.79	1.00
	**AC C20:4**	C_27_H_46_O_4_N_1_	448.3424	6.80	2.43	1.77	1.73
	**AC C20:3**	C_27_H_48_O_4_N_1_	450.3578	6.78	13.89 ^a^	2.87	2.17
	**AC C20:1**	C_27_H_50_O_4_N_1_	454.3891	6.71	0.71	1.25	11.19

^a^ the average normalized peak area for HGG is statistically significantly different from LGG, *p* < 0.05; ^b^ the average normalized peak area for IDHw is statistically significantly different from IDHm, *p* < 0.05; ^c^ the average normalized peak area for n-del is statistically significantly different from del, *p* < 0.05.

## Data Availability

Not applicable.

## Supplementary Materials

The following are available online. Figure S1. Correlation plot of short chain acylcarnitines and long chain acylcarnitines; Table S1. Detailed list of normalized peak areas in all samples; Table S2. Ratios of selected acylcarnitines and carnitine used to estimate the activity of enzymes related to the carnitine shuttle system; Table S3. Detailed description of samples included in the study; Table S4. Selection of sampling condition. Comparision of sampling on ice and in the room temperature. Table S5. Acylcarnitines which could be identified using LipidSearch.

## Sample Availability

Samples of the compounds are not available from the authors.
