# Supplementary material for: Profiling of Carnitine Shuttle System Intermediates in Gliomas Using Solid-Phase Microextraction (SPME)
Source: Molecules. 2021 Oct 10;26(20):6112. doi: 10.3390/molecules26206112 (PMC8540799; doi:10.3390/molecules26206112)
Supplement: Supplementary file 1 [file molecules-26-06112-s001.zip › molecules-1329195-supplementary.pdf]

Profiling of carnitine shuttle system intermediates in gliomas using solid-phase microextraction (SPME)

Joanna Bogusiewicz[1], Katarzyna Burlikowska[1], Karol Jaroch[1], Paulina Zofia Gorynska[1], Krzysztof Gorynski[1], Marcin Birski[2], Jacek Furtak[2], Dariusz Paczkowski[2#], Marek Harat[2,3], Barbara Bojko[1*]

1. Department of Pharmacodynamics and Molecular Pharmacology, Faculty of Pharmacy, Collegium Medicum in Bydgoszcz, Nicolaus Copernicus University in Torun, Bydgoszcz, Poland
2. Department of Neurosurgery, 10th Military Research Hospital and Polyclinic, Bydgoszcz, Poland
3. Department of Neurosurgery and Neurology, Faculty of Health Sciences, Collegium Medicum in Bydgoszcz, Nicolaus Copernicus University in Torun, Bydgoszcz, Poland

*Corresponding author: Barbara Bojko, bbojko@cm.umk.pl

Department of Pharmacodynamics and Molecular Pharmacology

Jurasza 2 Street, 85089 Bydgoszcz, Poland

# current affiliation: Department of Neurosurgery and Neurology, Jan Biziel University Hospital Collegium Medicum Nicolaus Copernicus University, Bydgoszcz, Poland

Figure S1. Correlation plot of short chain acylcarnitines and long chain acylcarnitines.

LCAC – long-chain-length acylcarnitine; SCAC – short-chain-length acylcarnitines

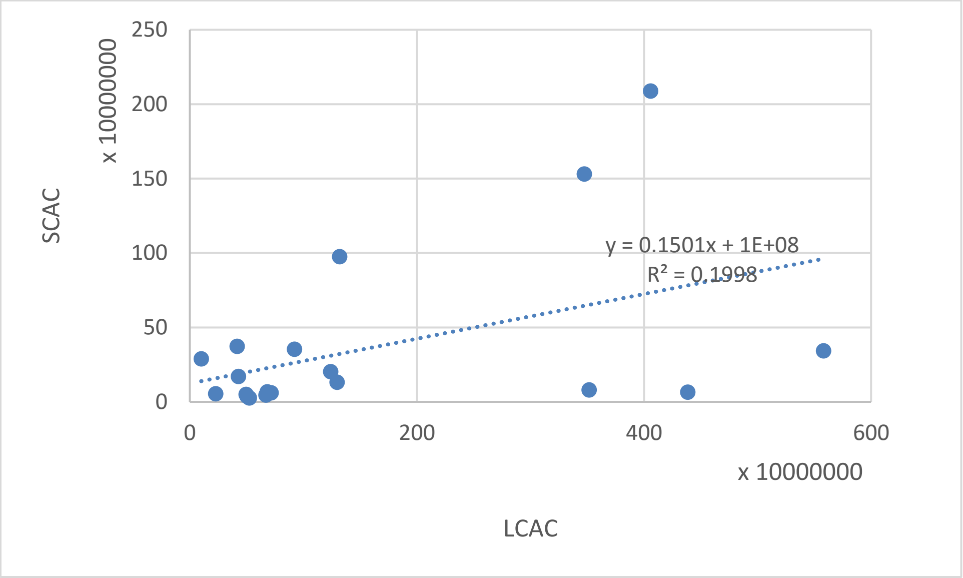

Table S1 Detailed list of normalized peak areas in all samples.

AC – acylcarnitine;

Number	102	104	108	110	111	112	117	120	122	125	128	129	130	131	132	133	135	136	138
**AcCa(2:0)**	0.46	0.70	0.58	0.56	0.59	0.76	0.73	0.63	0.57	0.89	0.68	0.27	0.59	0.62	0.48	0.43	0.67	0.64	0.78
**AcCa(3:0)**	0.01	0.04	0.06	0.08	0.06	0.03	0.14	0.06	0.01	0.02	0.03	0.07	0.05	0.03	0.08	0.02	0.08	0.09	0.03
**AcCa(4:0)**	0.18	0.07	0.12	0.08	0.09	0.10	0.06	0.09	0.17	0.05	0.17	0.12	0.16	0.18	0.16	0.19	0.10	0.09	0.10
**AcCa(5:1)**	0.01	0.01	0.00	0.01	0.01	0.00	0.00	0.00	0.01	0.01	0.01	0.01	0.01	0.01	0.00	0.01	0.01	0.00	0.00
**AcCa(5:0)**	0.34	0.18	0.24	0.27	0.25	0.12	0.08	0.21	0.24	0.03	0.11	0.53	0.19	0.17	0.29	0.35	0.15	0.19	0.08
**AcCa(6:0)**	0.74	0.78	0.73	0.61	0.15	0.73	0.32	0.14	0.56	0.21	0.60	0.23	0.47	0.38	0.39	0.29	0.52	0.21	0.44
**AcCa(8:0)**	0.19	0.21	0.18	0.23	0.30	0.17	0.32	0.32	0.27	0.36	0.33	0.39	0.31	0.33	0.44	0.39	0.30	0.35	0.31
**AcCa(9:0)**	0.00	0.00	0.00	0.00	0.01	0.00	0.03	0.03	0.00	0.00	0.00	0.01	0.01	0.00	0.01	0.00	0.01	0.01	0.00
**AcCa(10:1)**	0.01	0.00	0.02	0.05	0.09	0.00	0.19	0.19	0.01	0.08	0.01	0.05	0.02	0.03	0.03	0.08	0.03	0.11	0.06
**AcCa(10:0)**	0.06	0.01	0.07	0.11	0.45	0.09	0.14	0.32	0.16	0.35	0.06	0.33	0.19	0.26	0.13	0.23	0.15	0.32	0.18
**AcCa(12:1)**	0.04	0.00	0.07	0.06	0.13	0.01	0.04	0.08	0.05	0.10	0.02	0.03	0.01	0.02	0.02	0.01	0.07	0.03	0.05
**AcCa(12:0)**	0.07	0.02	0.07	0.09	0.17	0.03	0.05	0.11	0.06	0.14	0.07	0.06	0.06	0.02	0.08	0.02	0.12	0.05	0.08
**AcCa(14:2)**	0.01	0.00	0.03	0.07	0.05	0.00	0.02	0.06	0.03	0.05	0.00	0.03	0.01	0.00	0.03	0.01	0.06	0.02	0.04
**AcCa(14:1)**	0.15	0.05	0.16	0.11	0.14	0.07	0.09	0.12	0.13	0.15	0.12	0.12	0.09	0.04	0.07	0.04	0.17	0.07	0.12
**AcCa(14:0)**	0.13	0.08	0.10	0.15	0.07	0.08	0.07	0.08	0.09	0.12	0.10	0.06	0.11	0.09	0.10	0.05	0.09	0.07	0.10
**AcCa(16:1)**	0.16	0.09	0.08	0.05	0.08	0.08	0.11	0.07	0.16	0.08	0.09	0.14	0.09	0.19	0.05	0.22	0.08	0.10	0.09
**AcCa(16:0)**	0.20	0.33	0.22	0.26	0.15	0.34	0.29	0.23	0.24	0.18	0.29	0.23	0.30	0.35	0.40	0.26	0.15	0.30	0.24
**AcCa(18:2)**	0.04	0.04	0.02	0.02	0.03	0.05	0.06	0.04	0.03	0.03	0.05	0.05	0.02	0.03	0.03	0.08	0.03	0.06	0.05
**AcCa(18:1)**	0.20	0.29	0.19	0.16	0.14	0.30	0.24	0.15	0.21	0.12	0.26	0.27	0.22	0.25	0.18	0.30	0.12	0.24	0.18
**AcCa(20:4)**	0.00	0.02	0.05	0.02	0.01	0.02	0.03	0.01	0.00	0.03	0.00	0.01	0.03	0.00	0.02	0.01	0.07	0.02	0.03
**AcCa(20:3)**	0.00	0.00	0.00	0.00	0.01	0.00	0.00	0.01	0.00	0.00	0.00	0.01	0.02	0.00	0.00	0.01	0.02	0.02	0.01
**AcCa(20:1)**	0.00	0.08	0.00	0.00	0.02	0.02	0.00	0.01	0.00	0.00	0.01	0.00	0.04	0.00	0.01	0.00	0.00	0.03	0.01

Table S2. Ratios of selected acylcarnitines and carnitne used to estimate the activity of enzymes related to the carnitine shuttle system

AC – acylcarnitine; CV – coefficient of variation; del – presence of 1p/19q co-deletion; HGG – high-grade glioma; IDHm – IDH mutation, IDHw – IDH wildtype; LCAC – long-chain-length acylcarnitine; LGG – low-grade glioma; MCAC – medium-chain-length acylcarnitines; n-del – absence of 1p/19q co-deletion; SCAC – short-chain-length acylcarnitines

**Group**	**Compound**	**malignancy grade**						**IDH mutation status**						**1p/19q codeletion status**					
		**LGG**		**HGG**		**AREA RATIO (HGG/LGG)**	**t.test**	**IDHm**		**IDHw**		**AREA RATIO (IDHw/IDHm)**	**t.test**	**del**		**n-del**		**AREA RATIO (n-del/del)**	**t.test**
		**average**	**CV**	**average**	**CV**			**average**	**CV**	**average**	**CV**			**average**	**CV**	**average**	**CV**		
	**AcCa(2:0)**	0.668	21%	0.579	24%	**0.87**	0.191	0.620	30%	0.603	10%	**0.97**	0.803	0.639	26%	0.596	21%	**0.93**	0.532
	**AcCa(3:0)**	0.023	49%	0.067	44%	**2.89**	0.002[a]	0.039	100%	0.065	25%	**1.68**	0.074	0.036	126%	0.060	32%	**1.68**	0.116
**SCAC**	**AcCa(4:0)**	0.131	42%	0.112	34%	**0.85**	0.385	0.122	44%	0.115	31%	**0.94**	0.750	0.132	46%	0.111	29%	**0.84**	0.335
	**AcCa(5:1)**	0.007	38%	0.007	66%	**0.95**	0.841	0.008	46%	0.006	68%	**0.78**	0.349	0.008	47%	0.007	62%	**0.83**	0.472
	**AcCa(5:0)**	0.171	58%	0.235	52%	**1.38**	0.255	0.212	73%	0.211	27%	**1.00**	0.985	0.185	70%	0.227	49%	**1.22**	0.468
	**AcCa(6:0)**	0.571	37%	0.377	49%	**0.66**	0.051	0.469	47%	0.426	51%	**0.91**	0.677	0.420	43%	0.465	51%	**1.11**	0.673
	**AcCa(8:0)**	0.267	28%	0.320	22%	**1.20**	0.135	0.295	27%	0.307	24%	**1.04**	0.737	0.310	21%	0.295	28%	**0.95**	0.684
**MCAC**	**AcCa(9:0)**	0.000	265%	0.009	117%	**29.98**	0.045[a]	0.004	223%	0.008	126%	**2.12**	0.334	0.005	214%	0.007	143%	**1.37**	0.708
	**AcCa(10:1)**	0.020	147%	0.076	78%	**3.86**	0.032[a]	0.051	113%	0.060	97%	**1.18**	0.739	0.066	94%	0.049	112%	**0.74**	0.538
	**AcCa(10:0)**	0.142	88%	0.218	52%	**1.53**	0.193	0.182	62%	0.199	67%	**1.09**	0.767	0.199	48%	0.185	74%	**0.93**	0.815
	**AcCa(12:1)**	0.034	100%	0.051	66%	**1.50**	0.310	0.035	81%	0.055	69%	**1.56**	0.218	0.045	62%	0.045	86%	**0.99**	0.985
	**AcCa(12:0)**	0.061	69%	0.082	50%	**1.34**	0.300	0.056	67%	0.093	40%	**1.66**	0.047[b]	0.065	64%	0.079	53%	**1.22**	0.481
	**AcCa(14:2)**	0.013	160%	0.035	57%	**2.72**	0.034[a]	0.018	97%	0.036	67%	**1.94**	0.091	0.022	82%	0.029	84%	**1.33**	0.507
	**AcCa(14:1)**	0.102	46%	0.108	35%	**1.06**	0.761	0.097	45%	0.116	31%	**1.19**	0.321	0.105	45%	0.107	35%	**1.02**	0.920
	**AcCa(14:0)**	0.099	18%	0.088	30%	**0.89**	0.344	0.089	27%	0.096	25%	**1.08**	0.510	0.094	27%	0.091	26%	**0.96**	0.772
**LCAC**	**AcCa(16:1)**	0.119	39%	0.098	46%	**0.82**	0.345	0.130	38%	0.078	22%	**0.60**	0.008[b]	0.142	36%	0.084	26%	**0.59**	0.003[c]
	**AcCa(16:0)**	0.276	26%	0.253	27%	**0.92**	0.496	0.266	23%	0.256	31%	**0.96**	0.758	0.252	23%	0.267	28%	**1.06**	0.649
	**AcCa(18:2)**	0.040	25%	0.042	40%	**1.05**	0.784	0.045	32%	0.036	35%	**0.79**	0.143	0.045	39%	0.038	31%	**0.86**	0.366
	**AcCa(18:1)**	0.231	27%	0.200	27%	**0.87**	0.272	0.234	25%	0.186	26%	**0.79**	0.071	0.212	27%	0.211	29%	**1.00**	0.984
	**AcCa(20:4)**	0.010	131%	0.025	66%	**2.43**	0.062	0.014	85%	0.025	78%	**1.77**	0.157	0.013	108%	0.023	75%	**1.73**	0.227
	**AcCa(20:3)**	0.001	265%	0.009	89%	**13.89**	0.016[a]	0.003	147%	0.009	103%	**2.87**	0.093	0.003	148%	0.007	117%	**2.17**	0.282
	**AcCa(20:1)**	0.015	193%	0.011	117%	**0.71**	0.653	0.011	225%	0.014	95%	**1.25**	0.770	0.002	215%	0.019	121%	**11.19**	0.068

[a] the average peak area for HGG is statistically significantly different from LGG. $p<0.05$

[b] the average peak area for IDHw is statistically significantly different from IDHm. $p<0.05$

[c] the average peak area for n-del is statistically significantly different from del. $p<0.05$

Table S3 Detailed description of samples included in the study

AC – acylcarnitine; del – presence of 1p/19q co-deletion; F – female; HGG – high-grade glioma; IDHm – IDH mutation, IDHw – IDH wildtype; LGG – low-grade glioma; n-del – absence of 1p/19q co-deletion; M– male

Number	Age	Gender	Grade*	1p/19q co-deletion status	IDH1 mutation status	Location	Diagnosis**
102	33	F	LGG	del	IDHm	right frontal	Oligodendroglioma, IDH-mutant, and 1p/19q codeleted, Grade 2
104	32	M	LGG	n-del	IDHm	left temporo-parietal	Astrocytoma, IDH-mutant, Grade 2
108	52	F	HGG	n-del	IDHw	bifrontal	Glioblastoma, IDH-wildtype, Grade 4
110	64	M	HGG	n-del	IDHw	right temporal	Glioblastoma, IDH-wildtype, Grade 4
111	71	M	HGG	n-del	IDHw	ledt temporal	Glioblastoma, IDH-wildtype, Grade 4
112	40	F	LGG	n-del	IDHm	right frontal	Astrocytoma, IDH-mutant, Grade 2
117	31	M	HGG	del	IDHm	left parietal	Oligodendroglioma, IDH-mutant, and 1p/19q codeleted, Grade 3
120	50	M	HGG	n-del	IDHw	left frontal	Glioblastoma, IDH-wildtype, Grade 4
122	52	F	LGG	del	IDHm	left frontal	Oligodendroglioma, IDH-mutant, and 1p/19q codeleted, Grade 2
125	59	M	LGG	del	IDHm	right parietal	Oligodendroglioma, IDH-mutant, and 1p/19q codeleted, Grade 2
128	29	F	LGG	n-del	IDHw	left thalamus	Pilocytic astrocytoma, Grade 1
129	30	F	HGG	n-del	IDHm	left frontal	Astrocytoma, IDH-mutant, Grade 4
130	69	M	HGG	n-del	IDHw	bifrontal	Glioblastoma, IDH-wildtype, Grade 4
131	71	F	LGG	del	IDHm	left frontal	Oligodendroglioma, IDH-mutant, and 1p/19q codeleted, Grade 2
132	24	M	HGG	n-del	IDHw	fourth ventricle	Posterior fossa ependymoma, Grade 3
133	42	M	HGG	del	IDHm	right frontal	Oligodendroglioma, IDH-mutant, and 1p/19q codeleted, Grade 3
135	78	F	HGG	n-del	IDHw	right frontal	Glioblastoma, IDH-wildtype, Grade 4
136	60	M	HGG	n-del	IDHw	left fronto-parietal	Glioblastoma, IDH-wildtype, Grade 4
138	39	F	HGG	del	IDHm	right frontal	Oligodendroglioma, IDH-mutant, and 1p/19q codeleted, Grade 3

** Grade 1 and 2 tumors were included in the low-grade glioma (LGG) group; Grade 3 and grade 4 tumors, respectively, were included in the group of high-grade gliomas (HGG)*

***Tumor diagnoses were verified according to the 2021 WHO brain tumor classification*

Table S4 Comparision of sampling on ice and in the room temperature

For this purpose 5 C18 fibers were inserted in the mouse brains (region of hypthalmus, n=2). The sampling from one mouse was conducted on ice and from another one at room temperature. Sampling and analysis conditions were the same as in the experiment presented in Materials and Methods section. The obtained peak areas for all acylcarnitines were normalized on peak areas for SCAC, MCAC and LCAC.

AC – acylcarnitine; CV – coefficient of variation; F – sampling on ice; LCAC – long-chain-length acylcarnitine; MCAC – medium-chain-length acylcarnitines; RT – sampling in the room temperature; SCAC – short-chain-length acylcarnitines

Group	name	F							RT							ratio area [F/RT]	test t
		F1	F2	F3	F4	F5	average	CV	RT1	RT2	RT3	RT4	RT5	average	CV		
SCAC	AcCa(2:0)	0.729	0.826	0.788	0.723	0.648	**0.743**	9%	0.747	0.806	0.841	0.705	0.724	**0.764**	7%	**0.97**	0.600
	AcCa(3:0)	0.048	0.051	0.052	0.056	0.057	**0.053**	8%	0.059	0.048	0.046	0.088	0.081	**0.064**	30%	**0.82**	0.221
	AcCa(4:0)	0.095	0.075	0.094	0.119	0.127	**0.102**	21%	0.122	0.091	0.073	0.107	0.115	**0.101**	19%	**1.01**	0.964
	AcCa(5:1)	0.001	0.001	0.000	0.001	0.001	**0.001**	54%	0.001	0.001	0.001	0.001	0.002	**0.001**	32%	**0.78**	0.380
	AcCa(5:0)	0.128	0.047	0.066	0.100	0.167	**0.102**	47%	0.072	0.055	0.039	0.098	0.078	**0.068**	33%	**1.48**	0.200
MCAC	AcCa(6:0)	0.667	0.681	0.662	0.633	0.676	**0.664**	3%	0.626	0.584	0.638	0.578	0.586	**0.602**	5%	**1.10**	0.003[a]
	AcCa(8:0)	0.180	0.187	0.187	0.214	0.204	**0.194**	7%	0.213	0.229	0.186	0.221	0.230	**0.216**	8%	**0.90**	0.069
	AcCa(9:0)	-	-	-	-	-	-	-	-	-	-	-	-	-	-	-	-
	AcCa(10:1)	0.055	0.038	0.044	0.053	0.040	**0.046**	17%	0.050	0.045	0.037	0.047	0.049	**0.046**	11%	**1.00**	0.964
	AcCa(10:0)	0.098	0.094	0.107	0.100	0.079	**0.096**	11%	0.111	0.142	0.139	0.153	0.135	**0.136**	11%	**0.70**	0.001[a]
LCAC	AcCa(12:1)	0.021	0.006	0.019	0.016	0.023	**0.017**	38%	0.017	0.016	0.006	0.009	0.003	**0.010**	59%	**1.68**	0.119
	AcCa(12:0)	0.058	0.056	0.061	0.041	0.068	**0.057**	17%	0.060	0.058	0.030	0.041	0.032	**0.044**	32%	**1.29**	0.137
	AcCa(14:2)	0.014	0.002	0.012	0.007	0.013	**0.010**	52%	0.008	0.009	0.005	0.008	0.002	**0.006**	47%	**1.54**	0.229
	AcCa(14:1)	0.075	0.064	0.064	0.454	0.069	**0.145**	119%	0.071	0.064	0.345	0.052	0.035	**0.114**	115%	**1.28**	0.752
	AcCa(14:0)	0.100	0.098	0.103	0.060	0.104	**0.093**	20%	0.112	0.112	0.079	0.106	0.105	**0.103**	13%	**0.91**	0.392
	AcCa(16:1)	0.098	0.078	0.089	0.058	0.089	**0.082**	18%	0.094	0.104	0.058	0.086	0.077	**0.084**	21%	**0.98**	0.897
	AcCa(16:0)	0.235	0.290	0.251	0.142	0.261	**0.236**	24%	0.242	0.257	0.224	0.343	0.380	**0.289**	24%	**0.82**	0.217
	AcCa(18:2)	0.045	0.040	0.043	0.020	0.029	**0.035**	30%	0.043	0.041	0.023	0.035	0.020	**0.032**	33%	**1.09**	0.659
	AcCa(18:1)	0.209	0.226	0.219	0.129	0.212	**0.199**	20%	0.210	0.215	0.156	0.231	0.238	**0.210**	15%	**0.95**	0.634
	AcCa(20:4)	0.136	0.133	0.130	0.068	0.126	**0.119**	24%	0.140	0.114	0.066	0.078	0.090	**0.098**	30%	**1.21**	0.292
	AcCa(20:3)	0.001	0.001	0.003	0.000	0.001	**0.001**	79%	0.001	0.003	0.003	0.001	0.000	**0.002**	74%	**0.76**	0.578
	AcCa(20:1)	0.009	0.005	0.007	0.004	0.005	**0.006**	31%	0.002	0.007	0.006	0.011	0.018	**0.009**	67%	**0.68**	0.333

[b] the average normalized peak area for F is statistically significantly different from RT. $p<0.05$

Table S5 Aylcarnitines which could be identified using LipidSearch

AC – acylcarnitine; m/z – mass-to-charge ratio

AC	M/Z [M+H+]
AC C8:0	288.2169
AC C9:0	302.2326
AC C10:4	308.1856
AC C10:3	310.2013
AC C10:2	312.2169
AC C10:1	314.2326
AC C10:0	316.2482
AC C11:4	322.2013
AC C11:3	324.2169
AC C11:2	326.2326
AC C11:1	328.2482
AC C11:0	330.2639
AC C12:4	336.2169
AC C12:5	338.2326
AC C12:2	340.2482
AC C12:1	342.2639
AC C12:0	344.2795
AC C13:0	358.2952
AC C14:4	364.2482
AC C14:3	366.2639
AC C14:2	368.2795
AC C14:1	370.2952
AC C14:0	372.3108
AC C15:0	386.3265
AC C16:1	398.3265
AC C16:0	400.3421
AC C17:1	412.3421
AC C17:0	414.3578
AC C18:4	420.3108
AC C18:3	422.3265
AC C18:2	424.3421
AC C18:1	426.3578
AC C18:0	428.3734
AC C19:1	440.3734
AC C19:0	442.3891
AC C20:5	446.3265
AC C20:4	448.3421
AC C20:3	450.3578
AC C20:2	452.3734
AC C20:1	454.3891

AC C20:0	456.4047
AC C21:1	468.4047
AC C21:0	470.4204
AC C22:6	472.3421
AC C22:5	474.3578
AC C22:4	476.3734
AC C22:3	478.3891
AC C22:2	480.4047
AC C22:1	482.4204
AC C22:0	484.4360
AC C23:1	496.4360
AC C23:0	498.4517
AC C24:2	508.4360
AC C24:1	510.4517
AC C24:0	512.4673
